# A social-ecological assessment of food security and biodiversity conservation in Ethiopia

**DOI:** 10.1080/26395916.2021.1952306

**Published:** 2021-07-28

**Authors:** Joern Fischer, Arvid Bergsten, Ine Dorresteijn, Jan Hanspach, Kristoffer Hylander, Tolera S. Jiren, Aisa O. Manlosa, Patricia Rodrigues, Jannik Schultner, Feyera Senbeta, Girma Shumi

**Affiliations:** a Faculty of Sustainability, Leuphana University Lueneburg, Lueneburg, Germany; b Copernicus Institute of Sustainable Development, Utrecht University, Utrecht, The Netherlands; c Department of Ecology, Environment and Plant Sciences, Stockholm University, Stockholm, Sweden; d Social Sciences Department, Leibniz Centre for Tropical Marine Research (ZMT), Bremen, Germany; e Environmental Systems Analysis Group, Wageningen University and Research, Wageningen, The Netherlands; f Center for Environment and Development Studies, College of Development Studies, Addis Ababa University, Addis Ababa, Ethiopia

**Keywords:** Albert Norström, Agroecology, land sharing, land sparing, resilience, social-ecological systems, sustainability science, transdisciplinarity

## Abstract

We studied food security and biodiversity conservation from a social-ecological perspective in southwestern Ethiopia. Specialist tree, bird, and mammal species required large, undisturbed forest, supporting the notion of ‘land sparing’ for conservation. However, our findings also suggest that forest areas should be embedded within a multifunctional landscape matrix (i.e. ‘land sharing’), because farmland also supported many species and ecosystem services and was the basis of diversified livelihoods. Diversified livelihoods improved smallholder food security, while lack of access to capital assets and crop raiding by wild forest animals negatively influenced food security. Food and biodiversity governance lacked coordination and was strongly hierarchical, with relatively few stakeholders being highly powerful. Our study shows that issues of livelihoods, access to resources, governance and equity are central when resolving challenges around food security and biodiversity. A multi-facetted, social-ecological approach is better able to capture such complexity than the conventional, two-dimensional land sparing versus sharing framework.

## Introduction

With current species extinction rates up to 1000 times higher than prehistoric background rates (Barnosky et al. 2011) and a rise in the global number of food insecure people (FAO 2019), biodiversity conservation and food security are critical challenges. Because agriculture is a prerequisite for food security but also a main driver of biodiversity loss, food security and biodiversity conservation cannot be addressed separately. Scientists have addressed the intersection of food security and biodiversity conservation from different perspectives, spanning to different degrees framings from the natural and social sciences (Glamann et al. 2015; Kremen 2015). The dominant perspective is rooted in the natural sciences, and has emphasized issues of food availability (i.e. production) and land use trade-offs (Butsic et al. 2012; Zabel et al. 2019). Work taking this perspective often evokes the notions of land sparing, land sharing (Green et al. 2005; Phalan et al. 2011), and sustainable intensification (Tilman et al. 2011) (see Box 1 for a glossary of technical terms).

To better understand the complex interface between biodiversity conservation and food security, here, we demonstrate the practical implementation and value of a more comprehensive, but less widely applied social-ecological perspective. A social-ecological perspective on food security and biodiversity conservation recognizes that food security is not only a matter of food availability, but – following the Food and Agriculture Organization (FAO 2013) – also of access, stability and utilization; as well as the agency of local people to proactively shape the ways in which they obtain food (Fischer et al. 2017; Wittman et al. 2017). A social-ecological perspective further assumes that biodiversity has both intrinsic as well as instrumental values. Finally, it recognizes that landscapes are embedded within broader regional and global contexts, and are governed not by a single ‘decision-maker’ who acts on scientific evidence, but rather through a complex (and power-laden) suite of multi-level governance arrangements (Chappell et al. 2013; Crona et al. 2015; Ekroos et al. 2017).

**Figure 1.**
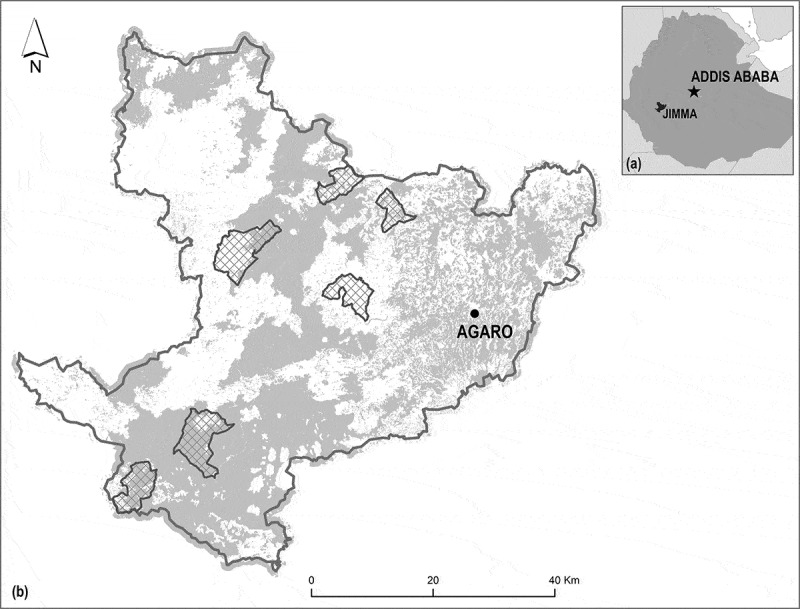
Study area location in (a) Jimma zone in southwestern Ethiopia, including (b) six local kebeles (hatched) within Jimma zone. Grey colour in (b) depicts woody vegetation, including both natural forest, coffee forest, and other areas of woody vegetation (e.g. in farmland)

In this paper, we summarize, synthesize, and reflect on five years of social-ecological research on food security and biodiversity conservation in southwestern Ethiopia. Ethiopia is the second most populous nation in Africa, and 80% of Ethiopians live in rural areas (Teller and Hailemariam 2011). Our study area is a smallholder-inhabited biodiversity hotspot, where issues of biodiversity conservation and food security are closely interlinked. The research was conducted in Jimma Zone, Oromia National Regional State, in southwestern Ethiopia, which consists of a mosaic of farmland and moist evergreen Afromontane forest (Figure 1). Southwestern Ethiopia is the centre of origin and diversity of *Coffea arabica*, and part of the Eastern Afromontane Biodiversity Hotspot (Mittermeier et al. 2011). Local livelihoods rely on smallholder agriculture, including crops and livestock; coffee and khat (a plant-based stimulant) are important cash crops. Food security in the area is low by international standards, and many households face seasonal food shortages.

Conceptually, our study built on the theoretical framework by Wittman et al. (2017), which posits that food security and biodiversity conservation are closely intertwined, and therefore cannot be meaningfully addressed in isolation of one another. Taking that perspective, the design of our study followed the theoretical principles of ‘integration by place, case and process’ previously recommended for place-based, inter- and transdisciplinary research (Sherren et al. 2010; Fischer et al. 2014). That is, we carried out separate and somewhat autonomous ecological and social sub-projects in the same study area; within this general place, we made sure the actual cases (e.g. households or communities) were shared across specific investigations; and we used numerous processes to integrate findings across the sub-projects, both within the research team (e.g. collaborative fieldwork, integrative papers) and with stakeholders (e.g. workshops, feedback sessions).

Following an overview of the overarching study design, we first provide a brief summary of key findings in the different sub-projects – which focused on biodiversity (woody plants, birds and mammals), food security and livelihood strategies, benefits and disbenefits of living with biodiversity, and challenges around equity and governance. Second, drawing on this summary, we highlight the most important social-ecological connections that emerged from the different sub-projects. Third, we summarize our efforts of and experiences with stakeholder engagement. We then discuss these insights in a broader social-ecological context, arguing that an approach similar to ours could be useful in many other settings around the world.

## Box 1. Glossary of technical terms

Coffee forest: Forest with wild Arabica coffee shrubs that is managed for coffee production at a range of intensities.

Governance: The structures and processes used by state and non-state actors to work together towards a certain outcome, such as sustainable development.

Immigration credit: A historical legacy effect whereby a certain patch has not yet reached its full potential in terms of its species richness, because more species will still colonise the patch in the future.

Instrumental value of biodiversity: The value of biodiversity in terms of its benefits to people, including both use values (e.g. trees provide firewood) and non-use values (e.g. trees may help to regulate floods).

Intrinsic value of biodiversity: The inherent value of biodiversity, regardless of possible benefits to people.

Land sparing: A conservation approach where some land is set aside specifically for biodiversity conservation, while the rest of the land is used for intensive agriculture.

Land sharing: A conservation approach where the same parcel of land is used to produce agricultural goods and conserve biodiversity.

Sustainable intensification: The increase of agricultural production per unit area in ways that are environmentally sustainable.

## Study design and overview of methods

Our research focused on six kebeles (smallest administrative unit in Ethiopia) in three woredas (districts) in Jimma Zone (Figure 1). The kebeles were chosen to span a diversity of social-ecological conditions but were not treated as replicates in a formal sense. While these six kebeles were the main focus, we also recognized higher-level social and ecological influences throughout our research. Within each kebele, variable numbers of sites or households were used, depending on the specific questions addressed. The methods used to assess biodiversity, food security, livelihoods and social equity, ecosystem services and disservices, and governance are summarized here only very briefly. Full details are given in the references to numerous publications that have outlined individual components summarized in this paper, and which are cited in the later sections of this paper where we summarize key findings. Much more detailed results, too, are available in the references provided. Similarly, a 150-page summary of all findings for stakeholders is also freely available online (Manlosa et al. 2020). The aim of the methods provided here therefore is to provide an overview of our work; readers are asked to consult the methodological references cited for a comprehensive step-by-step explanation of our methods.

### Methods used in the sub-projects

*Biodiversity distribution*. We surveyed woody plants in the forest (109 plots of 20 m x 20 m), in farmland (72 plots of 20 m x 20 m, embedded within 1 ha sites) and in homegardens (11 plots of 20 m x 20 m) distributed across the six kebeles introduced above. To analyse woody plant diversity, species were grouped into forest specialists, pioneers, and generalists. Data were analysed using constrained correspondence analysis, non-metric multidimensional scaling and generalized linear mixed effects models (Shumi et al. 2018, 2019b, 2020). Additionally, birds were surveyed in forest (n = 66 sites) and farmland (n = 83 sites), using point counts, and were grouped by ecological traits (Rodrigues et al. 2018). The forest dataset on birds was analysed using detrended correspondence analysis, canonical correspondence analysis and generalized linear mixed models (Rodrigues et al. 2018); farmland data are unpublished to date. Camera traps were used to survey mammals at the forest edge (25 sites across four kebeles, Rodrigues et al. 2019) and in the forest interior (95 sites in four kebeles, between 14 and 31 camera sites per kebele, Rodrigues et al. 2021). More than 500,000 photos were classified using ExiPRO^TM^ software. All pictures for the same species and triggered within a one-hour interval were considered the same event (Rodrigues et al. 2019). For the purpose of this paper, we used species recording rates (i.e. the proportion that a species was detected at a site over the duration of the survey at the site) as response variables in generalized linear models to explore individual species relationships with distance to the forest edge (see online supplementary material for details of the methods used specifically for this paper; detailed analyses are forthcoming).

*Food security, livelihood strategies, and social equity*. We surveyed 365 randomly selected households across the six kebeles. The survey covered (1) general household characteristics, (2) livelihoods, (3) capital assets, and (4) food security. Household livelihood strategies were identified and linked to capital assets and food security, using cluster analysis, principal components analysis and regression modelling (Manlosa et al. 2019). In addition, issues of social and gender equity, and underpinning social norms were examined using qualitative data from 20 focus group discussions and 45 interviews in three kebeles (Manlosa et al. 2018; Manlosa 2019). The data was subjected to qualitative thematic analysis using NVivo software.

*Benefits and disbenefits of living with biodiversity*. Smallholders experience both benefits (‘ecosystem services’) and challenges (‘ecosystem disservices’) associated with biodiversity (Ango et al. 2014). We surveyed 367 households about benefits (Schultner et al. 2021) and 150 households about the balance between benefits and challenges (Dorresteijn et al. 2017). In both cases, distinct beneficiary groups were identified through hierarchical clustering, and the clusters were tested for differences in socioeconomic and geographical characteristics. We also surveyed 180 households to specifically elicit the benefits obtained from woody plants, and summarized the numbers of woody plant species and their uses (Shumi et al. 2019a). This specific focus on woody plants was chosen because trees and shrubs are directly used by most local people; other elements of biodiversity may also be useful but not in the same immediate and direct ways (for example, wild mammals are not usually hunted here, unlike in many other parts of Africa). Finally, we combined our data on tree diversity and uses, and used regression modelling to visualise the relationship between woody species diversity and ecosystem services diversity (Shumi et al. 2020).

*Governance*. To assess governance structure and processes, interviews were conducted with 244 governmental, non-governmental and civil society stakeholders engaged in food security, biodiversity conservation, or both, from the local to the federal level (Jiren et al. 2018a, 2018b). Stakeholders were identified through snowball sampling, in which interviewees progressively identified additional stakeholders. The interviews focused on identifying collaborative links between stakeholders, and on governance challenges affecting food security and biodiversity. Social network analysis was applied to investigate and visualize patterns of stakeholder collaboration (Jiren et al. 2018a; Bergsten et al. 2019), while qualitative data on governance process challenges were analysed through content analysis (Jiren et al. 2021).

### Methodological approach to social-ecological integration

Our primary means of integrating the findings of the different sub-projects was inductive. That is, beyond the general framing outlined in the introduction and further explained by Wittman et al. (2017), we did not start with a pre-defined structure how the different sub-projects were going to be linked. This was a conscious methodological choice. Our approach was to probe the social-ecological system around general themes as defined by the different sub-projects; and then based on the findings of these, explore the links between different social and ecological issues. This approach to integration is both pragmatic (with different PhD projects taking place in parallel, and each project benefiting from a certain degree of autonomy) but also effectively prevents *ex ante* reductionism, which is common in existing work on the intersection of food and biodiversity. For example, the land sparing versus sharing framework specifically focuses on the relationship between agricultural yields and biodiversity – thereby pre-supposing that this relationship is of central importance in the particular setting under investigation. We specifically wanted to avoid strictly limiting ourselves to a relatively narrow set of questions or variables. An inductive approach centred around broad research themes (captured by the sub-projects) therefore was deemed appropriate. Inductive integration took place as an ongoing process throughout the research – a two-week immersion of all project members in the study area within the first few project months, weekly meetings of the whole research team, co-authoring papers across themes, and repeated in-depth exchange during and following extended periods of field work.

In addition to inductive integration, we also conducted participatory workshops with more than 30 local stakeholders to construct a shared social-ecological understanding of the study area in a formalized causal loop diagram. We also co-created with local stakeholders four scenarios depicting plausible futures for the study area. The causal loop diagram and scenarios are written up in a bilingual book targeting stakeholders (Fischer et al. 2018), as well as in a research paper (Jiren et al. 2020). We encourage readers to consult these publications, but do not reproduce the outcomes of these participatory processes in this paper – focusing here instead on the inductive integration of our findings by the research team.

## Summary of key findings in the sub-projects

This section summarizes the main results of the sub-projects introduced above. Readers are kindly asked to consult the references provided for further details.

*Biodiversity distribution*. We identified 158 species of trees and shrubs, 113 in the forest and 110 in farmland. In the forest, woody species composition was associated with altitude, forest type, coffee management intensity and distance from the forest edge. Forest specialist richness declined with coffee dominance (a proxy for the intensity of management), was lower in secondary forest, and increased with distance from the forest edge (Shumi et al. 2019b). In farmland, historical distance from the forest edge was positively correlated with the richness of generalist and pioneer species, indicating an immigration credit (Shumi et al. 2018).

Of 131 bird species, 112 were recorded in farmland and 76 in the forest; 13 species were endemic to Ethiopia and Eritrea. In the forest, different groups of birds responded differently to coffee dominance and landscape context. Insectivores and frugivores, in particular, were associated with the undisturbed forest interior, while less specialised species tolerated higher levels of coffee management and exposure to the forest edge (Rodrigues et al. 2018).

**Figure 2.**
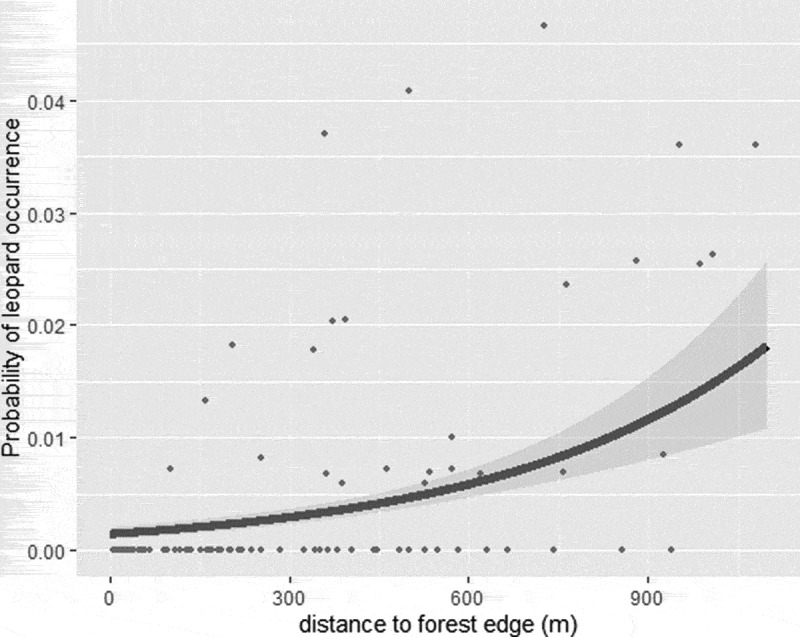
Probability of leopard occurrence in response to distance to the forest edge, while keeping elevation constant at its mean. Grey shading indicates the 95% confidence interval

For mammals, 32 species were recorded. These included the leopard (*Pantera pardus*) and hyena (*Crocuta crocuta*) as top predators, as well as the baboon (*Papio anubis*) and bushpig (*Potamochoerus larvatus*) as potentially crop raiding species (Rodrigues et al. 2021). Within the forest, the recording rate of the leopard increased with distance from the forest edge (Figure 2, Table S1a and S1b), whereas potentially crop-raiding species such as the baboon showed no response to distance from the edge (Tables S1a, S1b; Fig. S1).

*Food security, livelihood strategies and social equity*. Household food security was associated with diversified livelihood strategies. Livelihood strategies were strongly dominated by on-farm activities, while off-farm livelihood strategies played only a minor role in our study area. The most food secure households grew three different food crops as well as the cash crops coffee and khat, while households with the same cash crops but only one food crop were significantly less food secure (Manlosa et al. 2019). Livelihood strategies were underpinned by different sets of capital assets. The most food secure households had better access to a wider range of capital assets, including agricultural land and knowledge exchange with other farmers. Further, male-headed households and households with more educated heads were more food secure (Manlosa et al. 2019). Households headed by landless men were disadvantaged relative to those headed by better-off men (Manlosa 2019). Moreover, women were disadvantaged, with social norms around capital assets, decision-making processes, and allocation of activities constraining the ways in which they could pursue livelihoods. Despite some improvements following policy reforms, gender inequity remains widespread (Manlosa et al. 2018).

*Benefits and challenges of living with biodiversity*. Biodiversity and livelihoods were linked via ecosystem services and disservices. Local livelihoods critically relied on numerous ecosystem services from forest and farmland, which provided for food, fuel and shelter – including, for example, coffee, honey, fuelwood, tree products, medicinal plants, spices, and various crops (Dorresteijn et al. 2017; Shumi et al. 2019a; Schultner et al. 2021). Not all people benefitted equally, though, because access was mediated by factors such as household economy and land ownership – such that there were large differences between households (Schultner et al. 2021). Trees provided vital services, with 95 tree species used for 11 general purposes (Table 1) (Shumi et al. 2019a). Notably, the diversity of ecosystem services in a given plot of land was positively associated with the diversity of woody species in that plot, suggesting a synergy between biodiversity conservation and landscape multifunctionality (Shumi et al. 2020).

**Table 1.** Benefits provided by woody vegetation across the landscape. Trees and shrubs were particularly important elements of biodiversity. The benefits listed here were the most frequently noted uses for woody vegetation. For details, see Shumi et al. (2019a)

Benefit or use	Number of species used
House construction	52
Farm tools	42
Fuelwood	38
Honey production	37
Fence	36
Medicine	25
Coffee shade	23
Household utilities	21
Soil fertility	18
Animal fodder	17
Poles and timber	11

Ecosystem disservices were experienced mainly through wildlife that damaged crops and livestock, and this indirectly also affected people’s health, social relationships, education and incomes (Dorresteijn et al. 2017). The balance between ecosystem services and disservices differed markedly between households, in response to household location and socioeconomic status. Households close to the forest suffered greater disservices, and poorer households received lower forest benefits than wealthier ones (Dorresteijn et al. 2017).

*Governance*. Governance structure was found to be strongly hierarchical and top-down, and 80% of stakeholders were government organizations (Jiren et al. 2018a). Given this structure, power was concentrated at higher levels of the government system (especially the regional and national levels), while woreda and kebele level actors largely served the agendas set from above.

Seventy-one percent of the 244 stakeholders were simultaneously involved in the governance of food security and biodiversity conservation, suggesting a major opportunity to integrate the two sectors. However, in practice, many interactions were of a top-down nature. There were virtually no reported horizontal interactions of stakeholders across administrative boundaries – for example, neighbouring woredas (districts) did not interact at all but each woreda obtained its orders from the higher governance level and reported back to that level (i.e. the zone).

With regard to the management of interdependent sustainability issues, stakeholder collaboration was reasonably well developed and integrated around food and coffee production, but weaker and narrower for biodiversity conservation and for access provision to services such as finance, transportation, schools and markets (Bergsten et al. 2019). Further governance challenges included institutional misfit (e.g. lack of institutions that addressed the multiple dimensions of food security), problems of institutional interplay (e.g. lack of coordination), and incoherence in policy goals (e.g. between biodiversity conservation and agricultural development). These process-related challenges not only affected food security and biodiversity conservation in isolation, but also made their integration more challenging (Jiren et al. 2021).

## Synthesis of social-ecological connections

**Figure 3.**
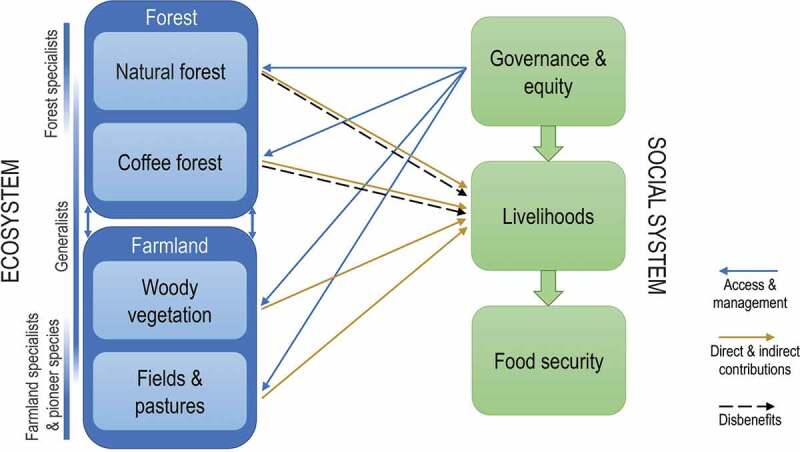
Synthesis of some of the most important social-ecological connections within the study landscape. Ensuring universal food security requires attending to all system components shown here; ensuring biodiversity conservation hinges primarily on land use governance

Inductive synthesis of the sub-projects summarized above showed numerous social-ecological connections within the landscape (Figure 3). Food security emerged as a central outcome of viable livelihood strategies. The viability of livelihood strategies of a given household, in turn, was heavily influenced by existing social inequities and governance arrangements. Equity challenges occurred around issues of access to land and ecosystem services, as well as around exposure to ecosystem disservices. Many such challenges were related to different levels of wealth or to gender. Power was concentrated with the government and exerted in a top-down fashion.

Livelihoods depended strongly on the natural resources offered by coffee forest, natural forest and farmland; including in particular many different species of trees. Both forest and farmland trees were critical for local livelihoods. Different elements of biodiversity, in turn, were associated with natural forest, coffee forest, and woody vegetation within the farmland. While the diversity of trees contributed in positive ways to local livelihoods, several species of mammals negatively impacted food security – forest patches harboured crop raiders (e.g. bush pig and baboon) and livestock predators (e.g. hyena and leopard; Figure 3).

**Figure 4.**
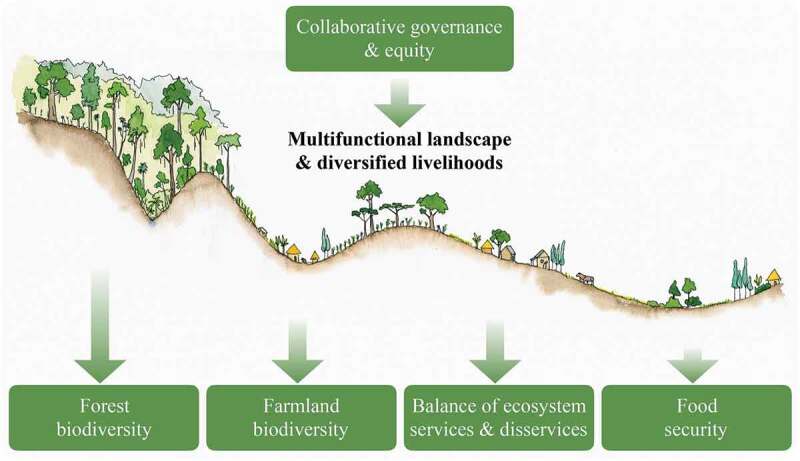
Schematic depiction of the role of landscape multifunctionality in southwestern Ethiopia. Large areas of undisturbed forest as well as a multifunctional landscape matrix appear to be important to harmonize biodiversity conservation and food security

Overall, our findings suggested that a multifunctional landscape, including natural forest, coffee forest, and heterogeneous farmland, underpinned both livelihoods and biodiversity (Figure 4). Agricultural yields did not emerge as the most critical constraint to local food security. In contrast, equity dimensions and crop raiding by wildlife were especially important. The social-ecological systems approach we applied thus highlights the complexity involved in harmonizing food security and biodiversity goals in a specific landscape. It reveals that these goals cannot be achieved through prescriptive and technical solutions alone, but will need to be negotiated and navigated by multiple stakeholders.

## Stakeholder engagement

We engaged stakeholders throughout our research. During a setup phase of six months, we introduced ourselves to 72 rural households as well as to representatives of all kebele (i.e. municipality) and woreda (i.e. district) administrations to discuss the feasibility of the research, refine research questions and adjust research methods. Over the following 3.5 years, we annually provided interim findings to households, schools, and kebele and woreda offices by distributing factsheets and posters summarizing our findings. Scenario planning engaged more than 30 stakeholder groups to develop a shared understanding of challenges and social-ecological interactions around food security and biodiversity conservation (Fischer et al. 2018; Jiren et al. 2020). In 2018 (the final year of the project), we organized outreach meetings including community- and administration-level workshops and a policy-oriented conference. We distributed scientific papers and outreach products including postcards aiming to stimulate discussion among stakeholders, and a bilingual book on plausible future scenarios for the study area (Fischer et al. 2018). An accessible synthesis book summarizing the entire project was published and disseminated in the study area in February 2020 (Manlosa et al. 2020).

With few exceptions, workshops and meetings with stakeholders included opportunities to obtain feedback. This feedback highlighted some gaps in our work – for example, government officers highlighted that beef fattening was a significant livelihood activity in some parts of the study area, which we had largely glossed over. However, stakeholders generally appreciated the collaborative approach we took, and some commented explicitly that our research was less ‘extractive’ than what they had previously experienced (Jiren et al. 2020). At present, as a follow-up to the work outlined here, we are trying to engage stakeholders in visioning processes seeking to identify plausible trajectories towards a sustainable future. This work is ongoing, although parts of it have been hampered by the Covid pandemic and political instability spilling over to our study area from other parts of Ethiopia.

## Discussion

Our findings offered new insights into the complex interrelationships between biodiversity conservation and food security. We discuss these findings with respect to implications for biodiversity conservation and food security, both in the context of our study area but also globally. Throughout the discussion, we contrast our approach with dominant narratives that focus on few variables and often prominently emphasize agricultural yields. Our central argument is that a social-ecological approach, as illustrated here, can provide a useful alternative to framings such as the land sparing versus sharing model, and could be adopted more widely.

Our ecological findings confirmed what conservationists have argued for decades – sensitive species require large, undisturbed areas. We agree with the likely general applicability of this take-home message also arising from several studies using the land sparing versus sharing framework (Green et al. 2005; Phalan et al. 2011). However, our findings strongly suggest that the frequently raised follow-up argument that if land sparing is superior for sensitive biodiversity, land sharing must be abandoned as an idea, is simply not meaningful. Instead, sustainable land management requires both relatively undisturbed areas as well as the sustainable management of farmland (Kremen 2015); which likely implies a multifunctional (and hence biodiverse) rather than industrialised agricultural matrix (Kremen and Merenlender 2018). We strongly suggest ecologists, policymakers, and land managers abandon the land sparing versus land sharing framework as a decision aid in complex social-ecological contexts, especially in smallholder-dominated landscapes.

Although increasing smallholder yields can be an important aspect for improving livelihoods in some instances (Snapp et al. 2010), the widespread fixation on yields among both ecologists and policymakers is likely a paradigmatic spillover of the green revolution, and of the neoliberal supremacy of ill-defined ‘efficiency’ as a valid goal in its own right. The clash apparent here is not unlike what Holling and Meffe (1996) termed the ‘pathology of natural resource management’ – which states that many social-ecological systems are not being managed as complex systems, but stabilized in ways that seek to maximize one particular aspect of system performance, such as crop production (Nyström et al. 2019). Existing framings around land sparing versus land sharing, sustainable intensification, or smallholder commercialization mirror this paradigm of ‘command and control’ (Holling and Meffe 1996). Moreover, the generation of simple, quantitative answers to highly complex problems generates the illusion of these answers being apolitical (Loconto et al. 2019) – when, in fact, framings that can be traced back to a green revolution logic tend to systematically (though often inadvertently) reaffirm existing power imbalances (Rosset and Altieri 2017). Engaged, social-ecological science, as advocated here, is unlikely to generate a simple definitive answer. However, it can provide a well-rounded set of insights that can be considered in multi-stakeholder discussions on how to navigate societal challenges (Fischer et al. 2015) – even, and especially, if opinions diverge among different stakeholders.

In concrete terms, our findings suggest that, in our study area, landscape multifunctionality is beneficial for smallholder livelihoods as well as for farmland biodiversity (Figure 4). Beyond the immediate benefits shown here, it is possible that multifunctional areas of farmland and forest also provide buffers for sensitive core areas (Hylander et al. 2013); an idea long recognized as valuable in conservation management (Noss and Harris 1986). While many industrial farming landscapes have evolved to maximize the provision of single commodity crops, it is increasingly clear that this shift has had many negative consequences. Among these are the heavy reliance on fossil-fuel and artificial-input dependent farming (Haberl et al. 2007; Kastner et al. 2014), loss of ecological function and resilience (Anderies et al. 2006; Tscharntke et al. 2008; Karp et al. 2012), but also loss of crop diversity (Khoury et al. 2014), and increasing disconnection of rural communities from their environments (Ives et al. 2017). Notably, the landscape we studied does not neatly fall into the simple schematic of a ‘pristine’ forest surrounded by ‘degraded’ farmland – rather, both forest and the agricultural mosaic supported different and complementary elements of biodiversity and ecosystem services (Gove et al. 2008, 2013; Engelen et al. 2016).

Globally, smallholder farming landscapes provide at least half of the world’s food (Graeub et al. 2016), and unlike industrial commodity production landscapes, typically generate food for local or national markets; thereby contributing much more directly to food security than, for example, intensive oil palm plantations, or soy monocultures that produce feed for distant livestock. Smallholder farming landscapes thus provide opportunities to harmonize food security and biodiversity conservation in ways that industrial farming landscapes have failed to do. On this basis, we argue that a critical challenge is not primarily to raise crop yields in the national balance sheets of smallholder-dominated countries, but rather to facilitate sustainable, universal and equitable access to sufficient, healthy food – as well as to other vital (natural) resources such as land. Indeed, we uncovered numerous challenges around equity and power imbalances, pertaining to government versus community, men versus women, and poor versus rich farmers. Science can play a vital role in helping to uncover such challenges, and can critically and empathetically engage with the local realities faced by people in a given location.

The social-ecological approach outlined here deviates from dominant approaches in conservation science. Instead of generating a simple model with definitive ‘evidence’ for a clearly defined ‘decision maker’, we collected both ecological and social puzzle pieces, and began to put these together in ways that can support multiple stakeholders to collaboratively navigate real-world complexity. Both our project as a whole as well as many individual papers combined several epistemological perspectives (Miller et al. 2008), for example by drawing on quantitative and qualitative research methods. Throughout, we considered each new finding as just one of many decision-relevant pieces of information; put together, the pieces provided an increasingly coherent understanding of the social-ecological system at hand. The findings generated, in turn, were shared widely, seeking to stimulate discussion and reflection among stakeholders. Especially towards the end of the project, we explicitly tried to facilitate deliberation among diverse stakeholders rather than communicate the empirical outcomes of simplified sub-questions as recommendations to a narrow set of decision-makers.

Notwithstanding the usefulness of this social-ecological approach, we are acutely aware that it still generated only a partial understanding of the studied system. For example, we did not specifically deal with human population growth in this paper, although it is a critical driver of both food insecurity and biodiversity loss (and we address it in more recent work; see Rodrigues 2020; Rodrigues et al. 2021). Similarly, the issues of climate change and declining soil fertility were frequently raised as important by local people, but our work only marginally addressed these topics. As such, even a social-ecological approach will, in practice, remain less than all-encompassing. However, it is more likely to capture important interrelationships than conventional, explicitly reductionist approaches – rather than merely deepening understanding around a few variables, a social-ecological approach seeks to deepen as well as broaden understanding of the interrelationships among many variables.

Finally, some but not all lessons derived from this study may be transferable to different settings. It is likely that the details will differ between locations – for example, what constitutes just and multifunctional land use in the drier parts of Ethiopia might be quite different from what we documented here. Nevertheless, the interconnections among good governance, social impacts, and conservation effectiveness are likely to be important in many if not all human-dominated settings (Bennett et al. 2019).

Engaged social-ecological research thus provides a tangible and implementable alternative to the imposition of placeless theories onto real-world communities. The identification of societal problems and discussions about future (sometimes conflicting) goals by different stakeholders is sometimes part of science and sometimes beyond it. Through engaging with real-world problems from many different perspectives in inter- and transdisciplinary ways, science can make a useful, long-term contribution to widely agreed societal goals, such as the improvement of biodiversity conservation and food security.

## Supplementary Material

Supplementary Materials
