# Supplementary material for: A social-ecological assessment of food security and biodiversity conservation in Ethiopia
Source: Ecosyst People (Abingdon). 2021 Jul 28;17(1):400–10. doi: 10.1080/26395916.2021.1952306 (PMC8352376; doi:10.1080/26395916.2021.1952306)
Supplement: Supplementary Materials [file TBSM_A_1952306_SM1216.pdf]

Supplementary Materials

S1. Methods - Mammal species responses

We investigated the responses of two apex predators (the leopard and hyena), and a crop-raiding species (the baboon) to distance to the forest edge. To this end, we excluded all cameras where sampling effort was less than 30 days (n=4) and we used species recording rates as the response variables, modelled as a combination of two vectors (successes, failures), with successes as the number of nights a species was detected at a site and failures as the number of nights the species was not detected at the same site.

We used generalized linear models with binomial distribution, and logit link to model species recording rates to distance to forest edge and elevation. Both predictor variables were scaled and centered prior to entering the models. Models were assessed for overdispersion as described in Zuur *et al.* (2013), and were corrected by using the quasibinomial distribution. Additionally, we checked for possible spatial autocorrelation in the model residuals, using Moran's I statistics (Tables S1a, S1b). Analyses and plots were performed in R software, version 3.4.2, using packages *stats*, *spdep* and *ggplot2*.

S2. Results

Table S1a. Results of generalized linear models, assessing the effect of distance to forest edge (distedge) and elevation on mammal species. Model fit is given by the proportion of deviance explained by each model. Codes for the significance levels: ***p < 0.001, **p < 0.01, *p < 0.05.

**species**	**estimate**	**[SE]**	**distedge**	**[SE]**	**elevation**	**[SE]**	**Model fit**
leopard	-5.712	[0.218] ***	0.621	[0.169] ***	0.438	[0.164] *	0.38
hyena[#]	-5.467	[0.489] ***	-0.355	[0.342]	0.257	[0.417]	0.15
baboon[#]	-2.522	[0.265] ***	-0.044	[0.128]	-0.377	[0.198]	0.12

[#] kebele was included in the model to correct for spatial autocorrelation in the residuals, kebele estimates not shown here.

Table S1b. Statistics for Moran's I spatial autocorrelation on the residuals of species models.

**species**	**observed**	**expected**	**standard error**	***P***
leopard	-0.064	-0.011	-1.283	0.902
hyena	0.015	-0.011	0.654	0.256
baboon	-0.053	-0.011	-1.019	0.845

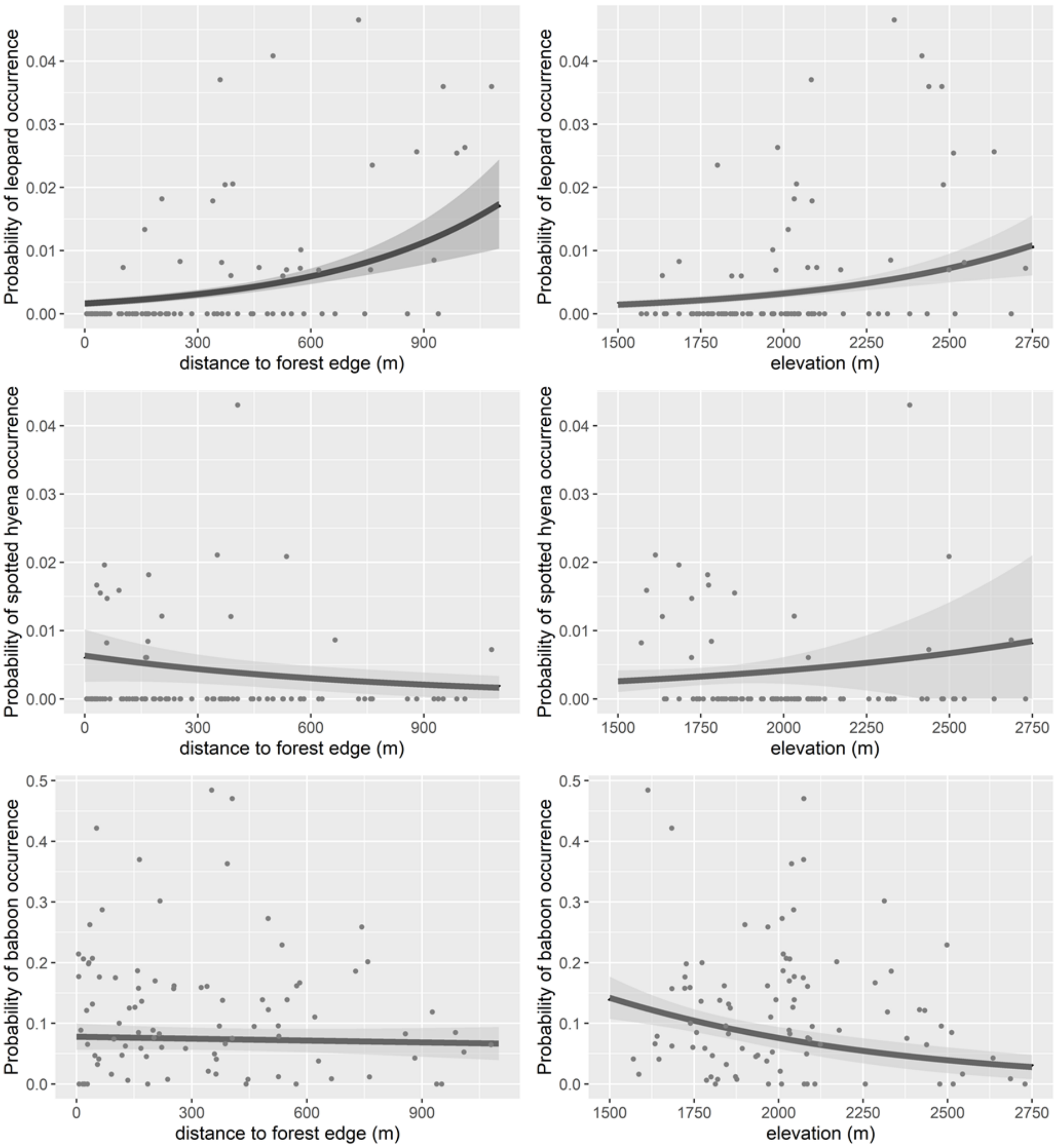

Fig. S1. Probability of species occurrence given distance to forest edge and elevation. Grey areas indicate 95% confidence intervals.

References

Zuur A. F., Hilbe J. M. and Ieno E.N. 2013. A beginner's guide to GLM and GLMM with R. Highland statistics.
